# A Gear-Driven Plantar Energy Harvester with Integrated Self-Sensing for Human Locomotion Recognition

**DOI:** 10.3390/s26165296

**Published:** 2026-08-21

**Authors:** Xinrui Wang, Weiqi Lin, Wenda Wang, Yang Yu, Moyue Cong, Yongzhuo Gao, Wei Dong

**Affiliations:** 1 State Key Laboratory of Robotics and Systems (HIT), Harbin Institute of Technology, Harbin 150001, China; 22b908055@stu.hit.edu.cn (X.W.); linweiqi@stu.hit.edu.cn (W.L.); wangwenda@stu.hit.edu.cn (W.W.); 15046962385@163.com (Y.Y.); 22b908052@stu.hit.edu.cn (M.C.); gaoyongzhuo@hit.edu.cn (Y.G.); 2 School of Mechatronics Engineering, Harbin Institute of Technology, Harbin 150001, China; 3 Weapon Equipment Research Institute, China South Industries Group Corp., Beijing 102202, China

**Keywords:** wearable energy harvester, electromagnetic induction generator, self-sensing, locomotion recognition, CNN–LSTM

## Abstract

Wearable electronic systems require compact and sustainable power sources together with reliable motion-sensing functions. This study presents a gear-driven plantar energy harvester that integrates biomechanical energy conversion with self-sensing locomotion recognition. The device converts low-frequency vertical foot loading into rotary motion through a wedge–lever transmission and amplifies the rotational speed using a multistage gear train with a total transmission ratio of 12. A one-way bearing enables directional power transmission during loading and prevents reverse rotation during recovery. The generated voltage serves both as the electrical output and as the sensing signal for locomotion recognition. Human-subject experiments were conducted under six locomotion modes: walking at 1, 2 and 3 m/s; running; ascending; and descending. Voltage signals were sampled at 2000 Hz and segmented into overlapping sequences. A CNN–LSTM model was used to extract local waveform features and temporal dependencies from the nonstationary signals. The model achieved an overall recognition accuracy of 98.8%, with most errors occurring between ascending and descending. The results demonstrate that a single plantar device can simultaneously harvest biomechanical energy and provide motion-related information, offering a compact solution for integrated energy harvesting and self-sensing in wearable systems.

## 1. Introduction

Wearable electronic systems have become increasingly important for health monitoring, rehabilitation, human–machine interaction, and activity recognition. However, their long-term operation remains strongly dependent on batteries, which require periodic charging or replacement and increase system weight and maintenance burden [1,2]. Human locomotion provides a continuously available biomechanical energy source that can potentially alleviate this dependence. In particular, repeated plantar loading during walking generates cyclic force and displacement, making the foot an attractive location for harvesting otherwise dissipated mechanical energy [3,4,5].

Different transduction mechanisms have been explored for plantar energy harvesting. Mechanical energy can be converted into electricity through electromagnetic, piezoelectric, and triboelectric mechanisms [6]. Piezoelectric and triboelectric devices can directly convert foot pressure or deformation into electrical output and have been widely investigated for flexible wearable systems [7,8,9,10,11]. Recent reviews have particularly highlighted the advantages of flexible triboelectric devices for simultaneous mechanical energy harvesting and self-powered wearable sensing [12]. In contrast, electromagnetic harvesters rely on relative motion between magnetic and conductive components and therefore generally require mechanical mechanisms to transform low-frequency plantar motion into sufficiently rapid generator motion [3,4,5]. Sharghi and Bilgen investigated pendulum-based electromagnetic generators for harvesting human walking energy [3], demonstrating the feasibility of rotational electromagnetic conversion from low-frequency body motion. Nevertheless, effective plantar electromagnetic harvesting remains strongly dependent on compact displacement conversion, directional transmission, rotational speed amplification, and reliable mechanical recovery.

Meanwhile, plantar devices are increasingly expected to provide not only energy but also information about human locomotion. Existing gait-monitoring systems typically employ pressure sensors, inertial measurement units, piezoresistive elements, or multimodal sensing arrays [13,14,15,16,17,18,19]. Zheng et al. developed a self-powered smart insole for high-resolution plantar-pressure mapping [7], while Tian et al. combined a smart insole with deep learning for multifunctional foot-health monitoring [20]. More recently, Wang et al. reported a wireless self-powered smart insole capable of gait monitoring and recognition [21]. These studies demonstrate the feasibility of extracting locomotion information from plantar signals. However, most such systems rely on dedicated sensing elements in addition to the energy-harvesting structure, increasing hardware complexity and system integration requirements.

An alternative approach is to directly exploit the electrical waveform generated by the energy harvester itself as a sensing signal. Plantar force, loading rate, contact duration, and gait frequency directly affect generator motion and consequently modify the amplitude and temporal characteristics of its voltage output. Previous studies have demonstrated self-powered motion sensing using triboelectric and other wearable transducers [9,20,21,22,23]. For example, triboelectric devices have been reported for human-motion monitoring and wearable self-powered sensing applications. Nevertheless, most existing self-sensing footwear systems rely on piezoelectric or triboelectric mechanisms, whereas mechanically amplified electromagnetic plantar harvesters have rarely been exploited simultaneously as both energy harvesters and locomotion-information sources [24].

Energy-harvesting voltage signals are inherently nonstationary and contain short-duration impact peaks, mechanical oscillations, and longer gait-dependent temporal patterns. CNN-based architectures are effective in extracting local waveform features, while LSTM networks can capture sequential dependencies in wearable activity signals [25,26,27]. More broadly, recent embedded monitoring studies have demonstrated that direct electrical waveform acquisition combined with CNN-based processing can provide effective state classification without relying on external sensing instrumentation [28]. This provides further support for extracting state-dependent information directly from electrical signals. A combined CNN–LSTM architecture is therefore suitable for distinguishing locomotion conditions from the raw voltage generated by the electromagnetic harvester.

In this study, a gear-driven plantar energy harvester with integrated locomotion self-sensing is proposed. Vertical foot loading is converted into rotational motion through a wedge–lever mechanism, while a one-way bearing and a multistage gear train provide directional transmission and rotational speed amplification for electromagnetic generation. The generated voltage is simultaneously used as the electrical output and as the input signal for locomotion recognition. Six locomotion modes, including walking at 1 m/s, walking at 2 m/s, walking at 3 m/s, running, ascending, and descending, were investigated using a CNN–LSTM classification model.

## 2. Materials and Methods

### 2.1. System Concept and Gait Energy Characteristics

**Figure 1:**
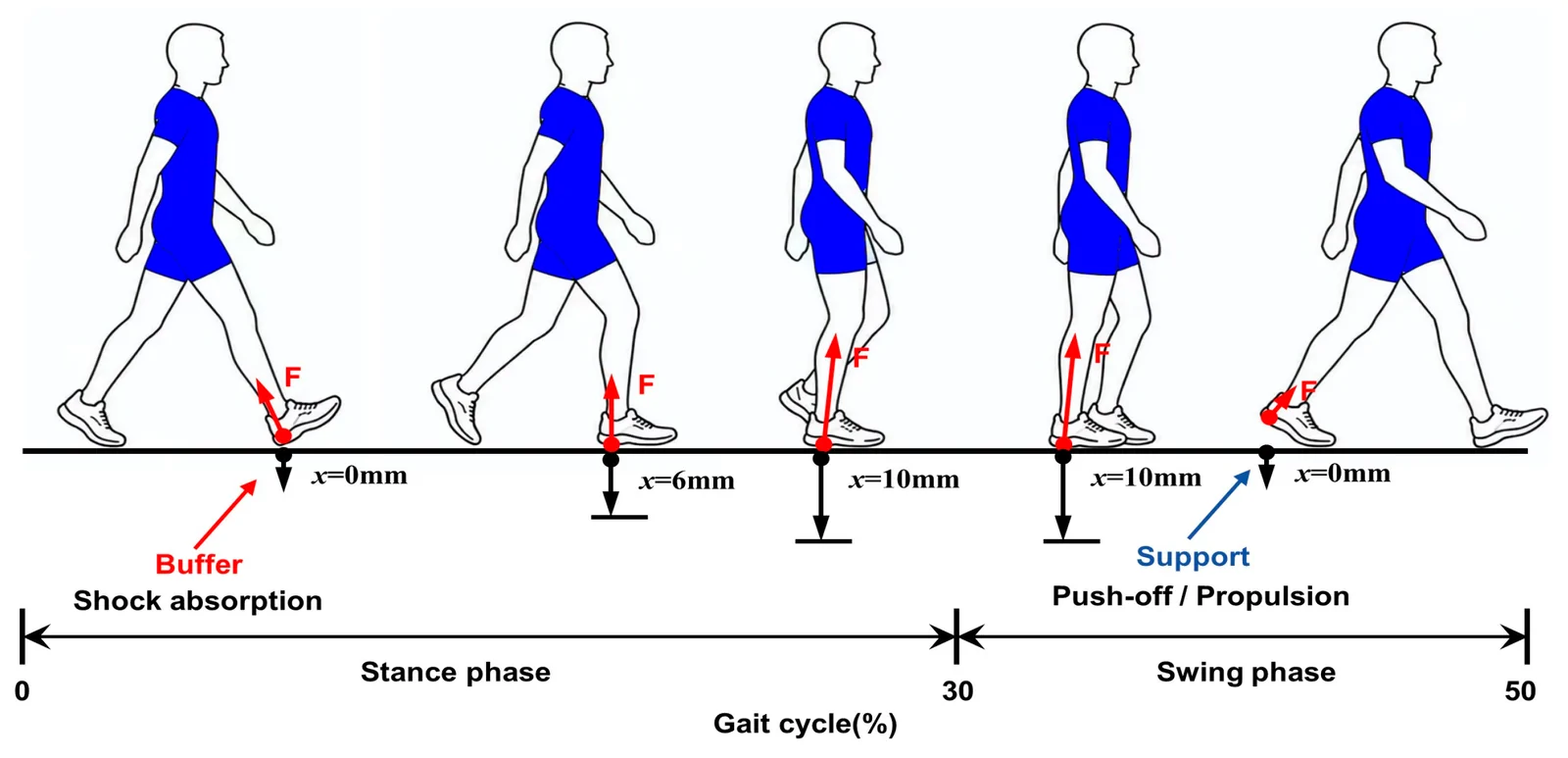
Human gait cycle and the main phases associated with plantar loading, including shock absorption, support, push-off, and swing.

Human locomotion involves periodic mechanical energy exchange between the body and the ground. During a normal gait cycle, the stance phase accounts for approximately 60% of the cycle, followed by the swing phase. At heel strike, the plantar region experiences a rapid vertical impact that provides both mechanical energy for harvesting and motion-dependent information for gait perception. The proposed system therefore places a compact energy-harvesting device beneath the plantar region to capture the vertical foot load. The human gait cycle and the period available for plantar biomechanical energy harvesting are illustrated in Figure 1.

**Figure 2:**
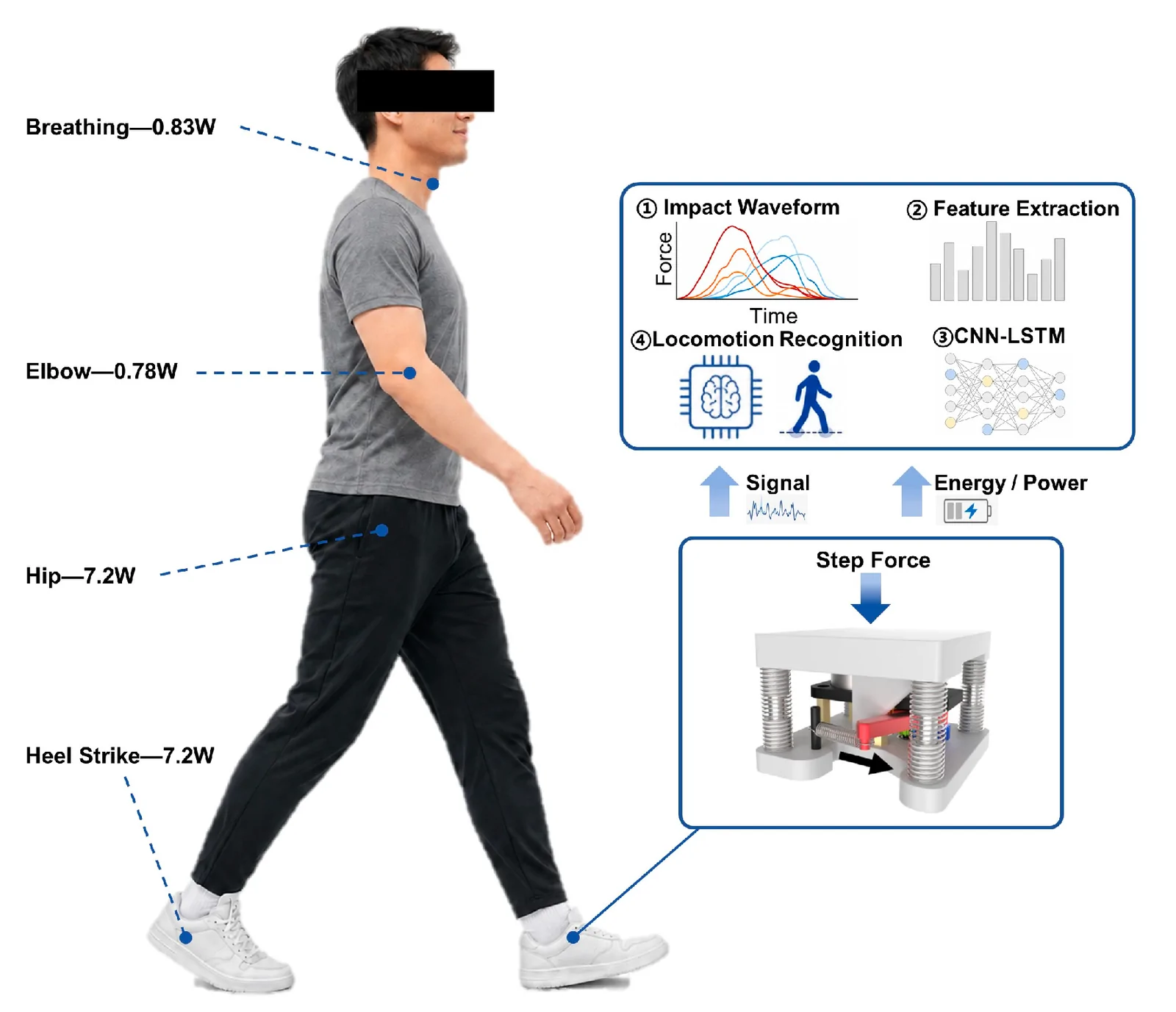
Overall concept of the proposed plantar energy-harvesting and self-sensing system.

The overall system integrates mechanical energy conversion, electrical generation, and locomotion recognition. Plantar loading drives the internal transmission mechanism and produces an electrical voltage through an electromagnetic generator. The generated electricity can be rectified and stored, while the original voltage waveform is simultaneously collected as a self-sensing signal. Since different locomotion modes produce different loading intensities, contact durations, and gait rhythms, their voltage waveforms contain distinguishable temporal characteristics that can be decoded using a deep-learning model. The overall concept of biomechanical energy harvesting and voltage-based locomotion recognition is shown in Figure 2. Plantar loading drives the electromagnetic generator to produce electrical energy, while the generated voltage is simultaneously used as the input signal for locomotion recognition.

### 2.2. Structure and Operating Principle of the Plantar Energy Harvester

The proposed plantar energy harvester mainly consists of a top cover, a baseplate, four compression springs, a wedge–lever transmission mechanism, an arc gear, a tension spring, a one-way bearing, a multistage gear train, and a miniature electromagnetic generator. The compression springs support the top cover and provide vertical recovery, while the tension spring restores the lever and arc gear after the plantar load is removed.

During the loading phase, the plantar force drives the top cover downward. The wedge converts the vertical displacement into the horizontal motion of the connecting rod, thereby rotating the lever and arc gear. The one-way bearing transmits the rotational motion to the multistage gear train, which amplifies the rotational speed before driving the electromagnetic generator.

**Figure 3:**
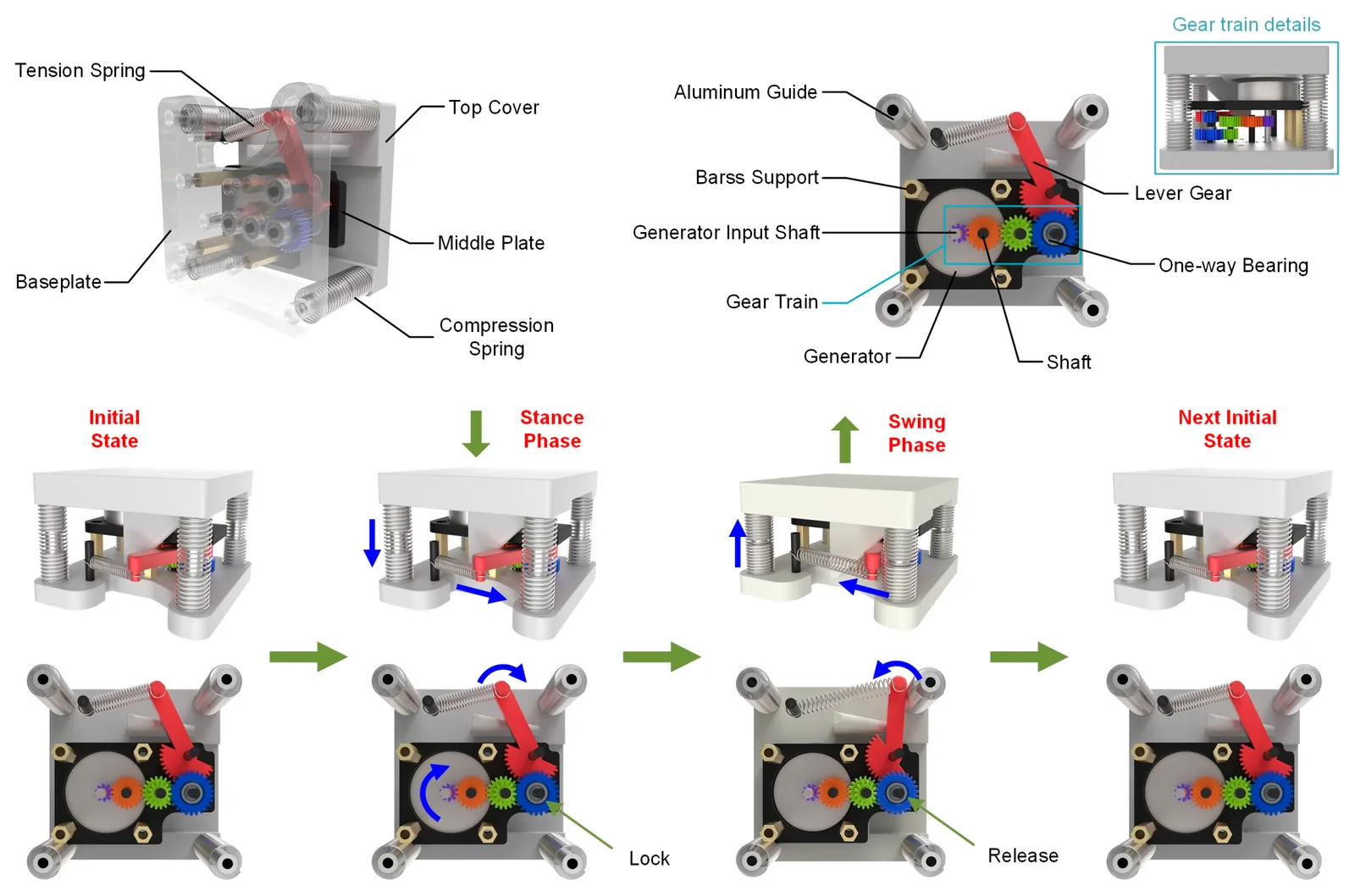
Structural configuration and operating process of the proposed plantar energy harvester.

During the unloading phase, the compression springs push the top cover upward, while the tension spring restores the lever and arc gear. The one-way bearing enters the overrunning state and disconnects the reverse motion from the gear train, thereby preventing reverse rotation of the generator. The structural configuration and loading–recovery process of the proposed harvester, as shown in Figure 3. Under plantar loading, downward motion of the top cover drives the wedge–lever mechanism and arc gear. After unloading, the compression and tension springs restore the mechanism to its initial configuration.

The multistage transmission consists of four gear pairs. The total speed-amplification ratio is determined by the product of the individual gear ratios.

(1) $$N=\frac{Z_{1}}{Z_{2}}\frac{Z_{3}}{Z_{4}}\frac{Z_{5}}{Z_{6}}\frac{Z_{6}}{Z_{7}}=\frac{36}{12}\times \frac{26}{13}\times \frac{20}{20}\times \frac{20}{10}=12$$

Accordingly, the rotational speed of the generator is related to the angular velocity of the arc gear by

(2) $$\omega_{g}(t)=N\omega_{\mathrm{in}}(t)$$

where *N* is the total speed-amplification ratio, $\omega_{\mathrm{in}}(t)$ is the instantaneous angular velocity of the arc gear, and $\omega_{g}(t)$ is the rotational speed of the generator. Therefore, the low-speed intermittent rotation generated by plantar loading is converted into a higher-speed rotational input for electromagnetic energy conversion.

**Figure 4:**
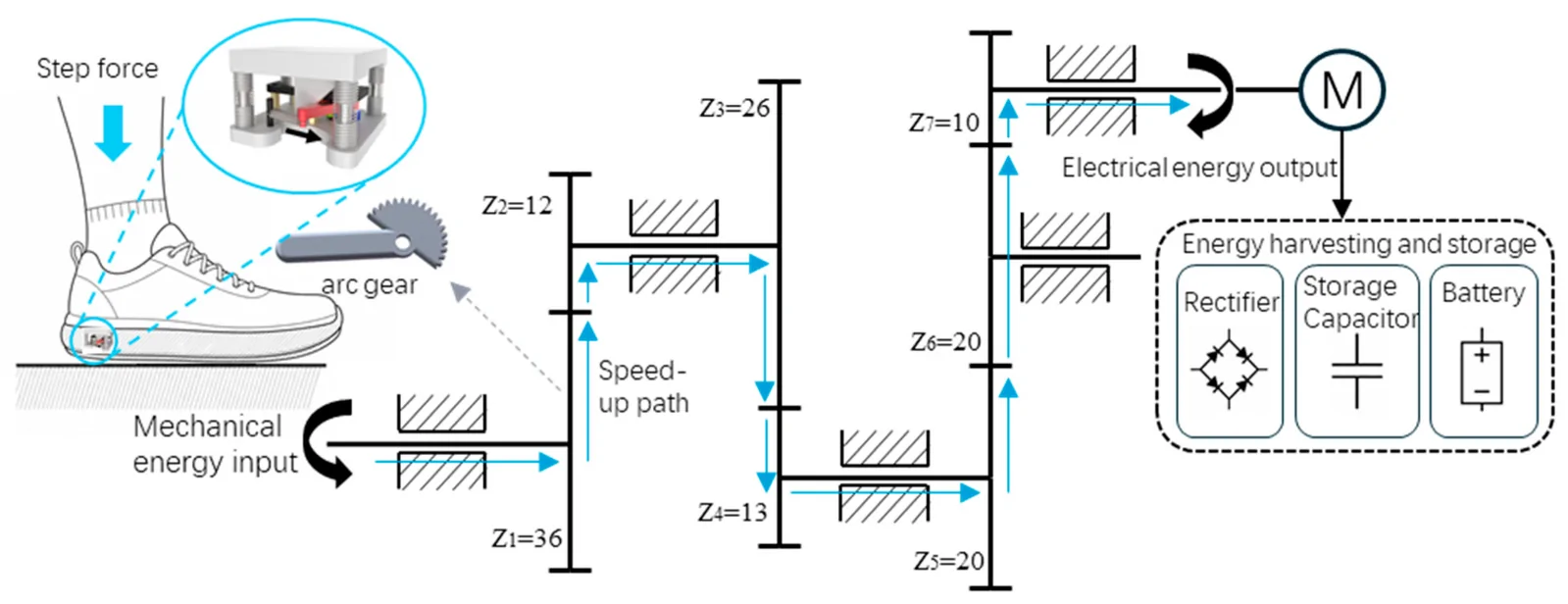
Multistage gear-transmission and electrical energy-management pathways of the proposed device.

The generator output can be connected to a power-management circuit consisting of a rectifier, a storage capacitor, and an external load or rechargeable battery. In the present study, this pathway represents a proposed power-management architecture and was not experimentally evaluated. The rectifier converts the alternating generator output into a unidirectional voltage, while the storage element accumulates the harvested electrical energy for subsequent use. The complete mechanical transmission and electrical energy-management pathways are summarized in Figure 4. The multistage gear train increases the rotational speed before driving the generator, and the generated electrical output is subsequently rectified and stored.

### 2.3. Kinematic and Electromechanical Modeling

To describe the mechanical energy conversion and electrical generation processes of the proposed plantar energy harvester, a coupled kinematic and electromechanical model was established. The vertical displacement of the wedge is denoted by *z*(*t*), the wedge angle by *α*, the horizontal displacement of the connecting rod by *x*(*t*), and the angular displacement of the arc gear by θ(t). Assuming that the connection point between the connecting rod and the arc gear moves along a circular trajectory with radius *L*, the geometric compatibility relationship can be expressed as

(3) x(t)=z(t)tanα=Lsinθ(t)

The angular displacement of the arc gear is therefore obtained as

(4) $$\theta (t)=\arcsin\left[\frac{z(t)\tan\alpha }{L}\right]$$

By differentiating Equation (4) with respect to time, the instantaneous angular velocity of the arc gear is expressed as

(5) $$\omega_{\mathrm{in}}(t)=\dot{\theta }(t)=\frac{\dot{z}(t)\tan\alpha }{\sqrt{L^{2}-{\left[z(t)\tan\alpha \right]}^{2}}}$$

Combined with the total speed-amplification ratio *N*, the rotational speed of the generator is

(6) $$\omega_{g}(t)=N\dot{\theta }(t)$$

The harvesting mechanism is further simplified as a single-degree-of-freedom system using *θ*(*t*) as the generalized coordinate. The equivalent inertia contains the translational inertia of the vertically moving components, the rotational inertia of the gear train, and the generator-rotor inertia reflected to the input shaft

(7) $$M_{\mathrm{eq}}(\theta )=m_{z}{\left(\frac{L\cos\theta }{\tan\alpha }\right)}^{2}+J_{\mathrm{gear}}+N^{2}J_{g}$$

where $m_{z}$ is the equivalent mass of the vertically moving components, $J_{gear}$ is the equivalent rotational inertia of the transmission system, and $J_{g}$ is the rotational inertia of the generator rotor. The $N^{2}J_{g}$ term indicates that the inertia of the high-speed generator rotor is amplified when reflected to the input shaft.

The elastic potential energy stored in the vertical compression springs and the lateral return spring is written as

(8) $$V(\theta )=\frac{1}{2}K_{z}{\left(\frac{L\sin\theta }{\tan\alpha }\right)}^{2}+\frac{1}{2}K_{\theta }L^{2}\sin^{2}\theta$$

where $K_{z}$ is the total stiffness of the vertical compression springs and $K_{\theta }$ denotes the equivalent stiffness contribution of the lateral return spring.

By applying the Lagrange equation, the nonlinear dynamic response of the harvesting system is governed by

(9) $$\begin{array}{l} M_{\mathrm{eq}}(\theta )\ddot{\theta }+\frac{1}{2}\frac{dM_{\mathrm{eq}}(\theta )}{d\theta }{\dot{\theta }}^{2}+\left(\frac{K_{z}L^{2}}{\tan^{2}\alpha }+K_{\theta }L^{2}\right)\sin\theta \cos\theta +C_{\mathrm{eq}}\dot{\theta }\\ =F_{\mathrm{foot}}(t)\frac{L\cos\theta }{\tan\alpha }-NT_{\mathrm{em}}(t) \end{array}$$

where $C_{eq}$ represents the equivalent mechanical damping, $F_{foot}(t)$ is the plantar excitation force, and $T_{em}(t)$ is the electromagnetic resisting torque. The second term on the left-hand side represents the nonlinear inertial effect caused by the configuration-dependent equivalent inertia.

The rotational motion of the generator induces a back electromotive force according to

(10) $$e(t)=K_{e}\omega_{g}(t)=K_{e}N\dot{\theta }(t)$$

where $K_{e}$ is the back-EMF constant.

Considering the coil inductance $L_{c}$, generator internal resistance $R_{in}$, and external load resistance $R_{L}$, the transient electrical response satisfies

(11) $$L_{c}\frac{di(t)}{dt}+\left(R_{\mathrm{in}}+R_{L}\right)i(t)=e(t)$$

The generated current produces an electromagnetic resisting torque

(12) $$T_{\mathrm{em}}(t)=K_{t}i(t)$$

where $K_{t}$ is the electromagnetic torque constant.

The instantaneous electrical power delivered to the external load and the harvested energy during one gait cycle are calculated as

(13) $$P_{\mathrm{out}}(t)=i^{2}(t)R_{L}$$

(14) $$E_{\mathrm{step}}=\int_{0}^{T_{\mathrm{step}}}i^{2}(t)R_{L}dt$$

The established model describes the complete energy-conversion process from vertical plantar displacement to arc-gear rotation, rotational speed amplification, electromagnetic generation, and electrical energy output. It also reveals that the mechanical response of the device is jointly governed by the nonlinear transmission geometry, reflected rotational inertia, elastic restoring force, mechanical damping, and electromagnetic load. These expressions provide a theoretical framework for evaluating electrical power and harvested energy; however, systematic experimental power and efficiency characterization was beyond the scope of the present study. The model was not used for formal numerical optimization of the present prototype; the current structural dimensions and gear configuration were mainly determined by structural feasibility, available components, and wearable-size constraints. Instead, the model was used to identify the principal parameters governing future optimization, including the transmission ratio, spring stiffness, reflected inertia, mechanical damping, and electrical load. Quantitative model validation was not performed because plantar force, displacement, loading rate, and generator response were not synchronously measured during the wearable experiments. Future controlled loading tests will therefore incorporate synchronized measurements of mechanical input and electrical output to enable quantitative validation using peak error, RMSE, and correlation coefficients.

### 2.4. Prototype Fabrication and Experimental Setup

A prototype of the proposed plantar energy harvester was fabricated based on the mechanical configuration described above. The device integrates a top cover, a baseplate, four vertical compression springs, a wedge–lever transmission mechanism, an arc gear, a one-way bearing, a multistage gear train, a return spring, and a miniature electromagnetic generator. The main structural components were fabricated using polymer 3D printing and metal additive manufacturing, while commercial gears, bearings, springs, shafts, and fasteners were used to assemble the transmission system. The prototype had a square footprint of 70 mm × 70 mm, a thickness of 50 mm, and a total mass of 138 g.

The four compression springs were symmetrically arranged between the top cover and the baseplate to support the external load and restore the initial height of the device after each step. The wedge–lever mechanism was installed beneath the top cover to convert vertical plantar displacement into angular motion of the arc gear. The multistage gear train and generator were mounted inside the supporting frame to reduce the lateral dimensions of the device and maintain stable engagement during repeated loading. The one-way bearing was connected between the input mechanism and the gear train to ensure directional transmission during the loading phase and to isolate the reverse motion during mechanical recovery.

For wearable testing, the prototype was mounted beneath the plantar region of the participant’s foot, as shown in Figure 4. The device was positioned to receive the vertical load generated during heel strike and the subsequent stance phase. A supporting fixture was used to maintain the relative position between the foot and the device during continuous locomotion while minimizing interference with the natural gait pattern.

**Figure 5:**
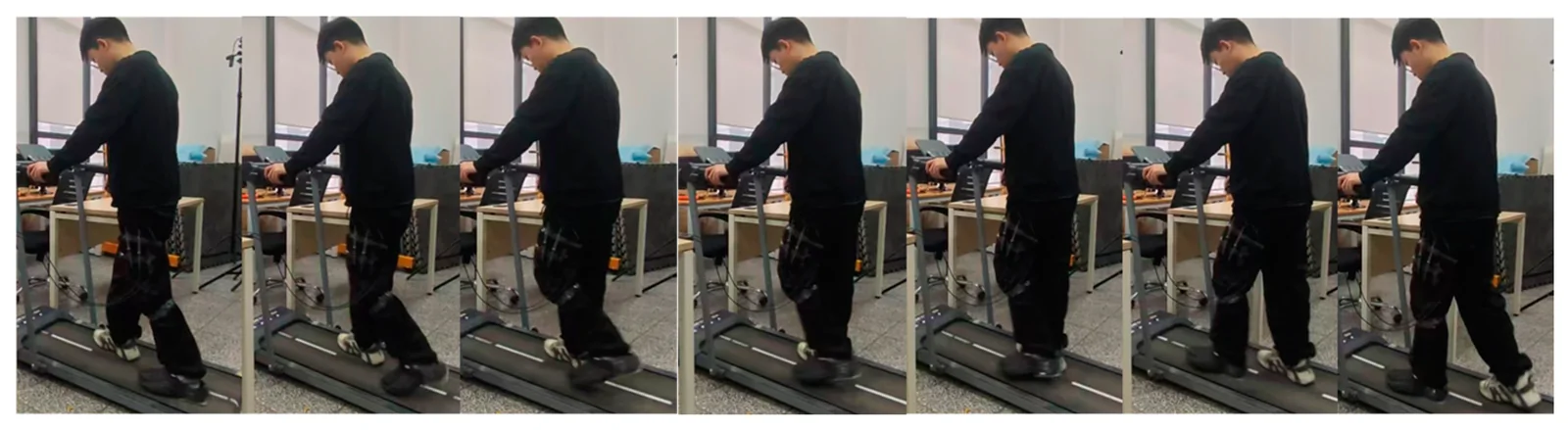
Sequential images of the wearable plantar energy harvester during treadmill locomotion.

Level walking and running experiments were conducted on a treadmill. The walking speeds were set to 1, 2, and 3 m/s, while the running speed was set to 5 m/s. Stair-ascent and stair-descent experiments were conducted on a fixed staircase under controlled laboratory conditions. Before formal data collection, each participant was allowed to practice the prescribed movements to become familiar with the device and experimental procedure. Representative sequential images of the wearable experiment are shown in Figure 5. The images demonstrate the repeated loading and recovery of the plantar device during continuous locomotion.

The electromagnetic and piezoelectric voltage signals were simultaneously recorded at 2000 Hz using a LOTO OSCA02 virtual oscilloscope connected to a computer. The sampling rate was sufficient to capture the transient peaks and oscillatory responses induced by plantar impact and mechanical transmission. All experiments were conducted under consistent laboratory conditions. The mechanical structure was inspected before each trial to ensure proper gear engagement, spring recovery, and electrical connection. The recorded voltage signals were subsequently used for both energy-output analysis and locomotion-mode recognition. For electrical power characterization, the electromagnetic generator was connected to an external resistive load of 60 Ω. The output voltage across the load was measured during running at 5 m/s, and the corresponding electrical output power was determined. A preliminary cyclic durability test was also performed by subjecting the assembled prototype to 10,000 repeated loading–unloading cycles in the plantar compression direction. The transmission mechanism was inspected before and after cycling for gear engagement, one-way transmission, structural damage, jamming, and recovery behavior.

### 2.5. Data Acquisition and Dataset Construction

To evaluate the feasibility of using the generated voltage as a self-sensing signal, human-subject experiments were conducted under different locomotion conditions. Three healthy participants with no reported history of musculoskeletal disorders, neuromuscular diseases, or gait-related impairments were recruited. Before data collection, all participants were informed of the experimental procedures and allowed sufficient time to become familiar with the wearable device and prescribed movements. Written informed consent was obtained from each participant prior to the experiments.

The participants performed six representative locomotion modes, including walking at 1 m/s, walking at 2 m/s, walking at 3 m/s, running, ascending, and descending. These modes were selected because they produce different plantar loading intensities, contact durations, impact frequencies, and gait rhythms. Consequently, the corresponding generator voltage signals exhibit distinguishable waveform amplitudes, temporal intervals, and transient characteristics.

The locomotion classes are defined as

(15) $$Y=\left\{y_{1},y_{2},y_{3},y_{4},y_{5},y_{6}\right\}$$

For each participant, every locomotion mode was continuously recorded for 200 s and repeated twice. A rest interval of at least 15 min was provided between repeated trials to reduce the influence of fatigue and short-term adaptation. During each trial, the voltage generated by the plantar energy harvester was recorded at a sampling frequency of 2000 Hz. The relatively high sampling frequency preserved the rapid voltage peaks and oscillatory responses produced by plantar impact, gear engagement, and generator rotation.

To avoid potential data leakage caused by overlapping sliding windows, the raw recordings were first divided into training, validation, and testing subsets according to participant and acquisition trial. Subsequently, sliding-window segmentation was independently performed within each subset. Therefore, samples originating from the same continuous recording were not distributed across different datasets. To further evaluate subject-independent generalization, leave-one-subject-out (LOSO) cross-validation was additionally performed. In each fold, all recordings from one participant were exclusively used for testing, while the data from the remaining two participants were used for model training and validation. Dataset partitioning was completed before sliding-window segmentation to prevent overlapping sequences from the same recording from appearing in different subsets.

The recorded voltage signals reflect the coupled mechanical and electrical responses of the device. Walking at different speeds changes the interval between consecutive plantar impacts and the magnitude of the applied load. Running generally produces stronger impacts and shorter contact durations, whereas ascending and descending generate different vertical loading sequences and body-weight transfer patterns. These differences provide the physical basis for locomotion-mode recognition using the generated voltage signal.

The continuous voltage signals were segmented using an overlapping sliding-window strategy. The window length was set to 2048 sampling points, corresponding to 1.024 s, while the sliding step was set to 128 sampling points, corresponding to 0.064 s. The (*k*)-th segmented voltage sample is expressed as

(16) $$X_{k}=\left\{v(kS),v(kS+1),\ldots ,v(kS+W-1)\right\},\;\;W=2048,\;S=128$$

where v(·) denotes the recorded voltage sequence, *W* is the window length, and *S* is the sliding step.

Each segmented sequence was assigned the label of the locomotion mode performed during the corresponding recording interval. The selected window length retained sufficient temporal information related to plantar loading and gait rhythm, while the overlapping segmentation generated samples from different phases of the locomotion cycle. This dataset-construction method enabled the recognition model to learn both local impact features and the temporal evolution of the voltage waveform.

Before model training, the segmented voltage samples were organized according to their locomotion labels and used as the input dataset for the CNN–LSTM classifier. The raw voltage sequences were directly used for feature learning, avoiding the need for manually designed time-domain or frequency-domain descriptors.

### 2.6. CNN–LSTM-Based Locomotion Recognition

**Figure 6:**
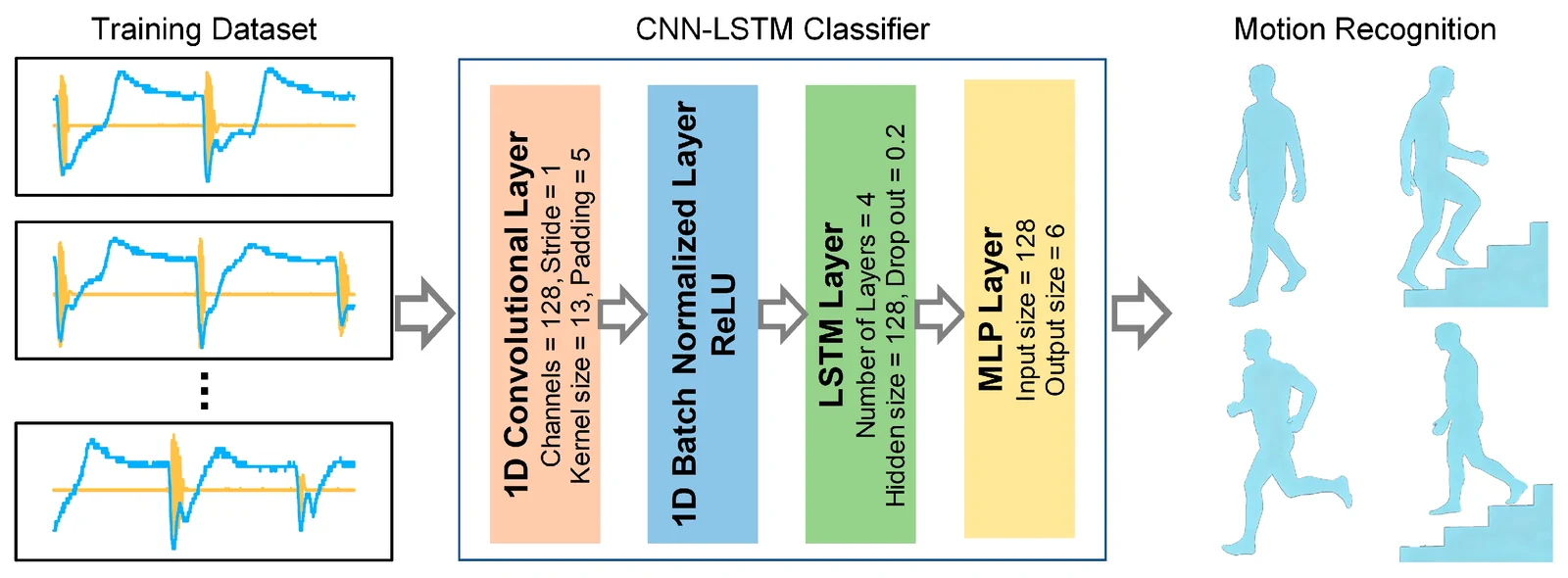
CNN–LSTM-based locomotion-recognition framework using voltage signals generated by the plantar energy harvester.

A CNN–LSTM model was developed to recognize the six locomotion modes directly from the segmented voltage sequences. The generated voltage signals contain both local waveform characteristics and long-term temporal dependencies. Local characteristics, including impact peaks, oscillatory responses, amplitude variations, and rapid waveform transitions, are mainly associated with plantar loading and mechanical transmission. Temporal characteristics, including gait rhythm, impact intervals, and step-to-step waveform evolution, reflect the sequential differences among locomotion modes. Therefore, the CNN and LSTM modules were combined to extract complementary spatial and temporal features from the voltage signals. The overall locomotion-recognition framework is illustrated in Figure 6. The continuous voltage signal is first segmented into fixed-length sequences, after which local waveform features and temporal dependencies are extracted using the CNN and LSTM modules, respectively. The final classifier outputs one of the six locomotion modes.

Each segmented voltage sequence was first input into a one-dimensional convolutional layer with 128 output channels, a kernel size of 13, a stride of 1, and a padding size of 5. The convolutional layer automatically extracted local discriminative patterns from the raw voltage waveform. A one-dimensional batch-normalization layer was subsequently applied to reduce the influence of amplitude variations among different trials and participants, followed by a rectified linear unit activation function to introduce nonlinear representation capability. The convolutional feature extraction process can be expressed as

(17) $$H_{\mathrm{CNN}}=\mathrm{ReLU}\left[\mathrm{BN}\left(\mathrm{Conv}1D(X)\right)\right]$$

The extracted feature sequence was then passed into four stacked LSTM layers. Each LSTM layer had a hidden size of 128, and a dropout rate of 0.2 was introduced to reduce overfitting. Through the input, forget, and output gates, the LSTM module selectively retained motion-related temporal information while suppressing irrelevant signal fluctuations. This enabled the model to capture gait periodicity, impact timing, and the sequential evolution of the voltage response.

The final temporal representation generated by the LSTM module was input into a multilayer perceptron classifier. The input dimension of the classifier was 128, and the output dimension was 6, corresponding to walking at 1 m/s, walking at 2 m/s, walking at 3 m/s, running, ascending, and descending. The classifier output was converted into class probabilities using the Softmax function

(18) $$p_{c}=\frac{\exp(o_{c})}{\sum_{j=1}^{6}\exp(o_{j})},\;\;c=1,2,\ldots ,6$$

where $o_{c}$ is the output score of the *c*-th locomotion class, and $p_{c}$ is the corresponding predicted probability.

The locomotion class with the highest predicted probability was selected as the final recognition result. The model was trained using the multiclass cross-entropy loss function

(19) $$L=-\frac{1}{B}\sum_{b=1}^{B}\sum_{c=1}^{6}y_{b,c}\ln p_{b,c}$$

where *B* is the batch size, $y_{b,c}$ is the ground-truth label, and $p_{b,c}$ is the predicted probability of the *c*-th class.

The model was implemented using PyTorch 2.8. The initial learning rate was set to 1 × 10^−3^, and the minimum learning rate was set to 1 × 10^−6^. A ReduceLROnPlateau scheduler was employed to automatically decrease the learning rate when the monitored training performance no longer improved. When the learning rate reached the minimum value and remained unchanged for 20 consecutive epochs, the training process was terminated.

The training and evaluation were conducted on a workstation equipped with a 13th-generation Intel Core i7-13700KF CPU, an NVIDIA GeForce RTX 4080 GPU, 16 GB RAM, and a 64-bit operating system. After training, the model received a fixed-length voltage sequence as input and output the corresponding locomotion class, enabling end-to-end recognition without manually designed time-domain or frequency-domain features. The CNN–LSTM model was trained and evaluated offline on the workstation and was not powered by the energy harvester.

## 3. Results

### 3.1. Voltage Responses Under Different Locomotion Modes

**Figure 7:**
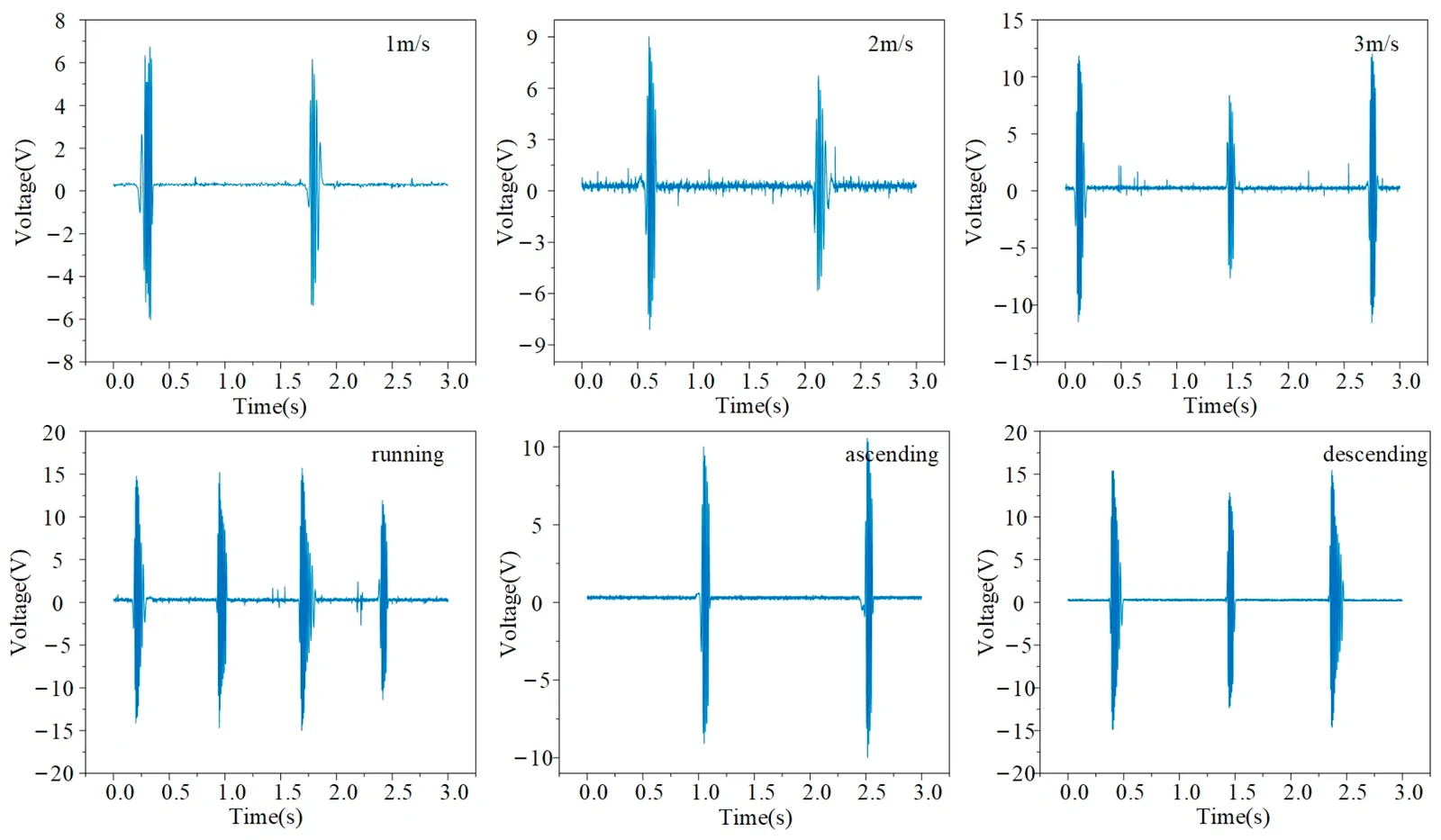
Representative electromagnetic voltage responses under six locomotion modes: walking at 1 m/s, walking at 2 m/s, walking at 3 m/s, running, ascending, and descending.

**Figure 8:**
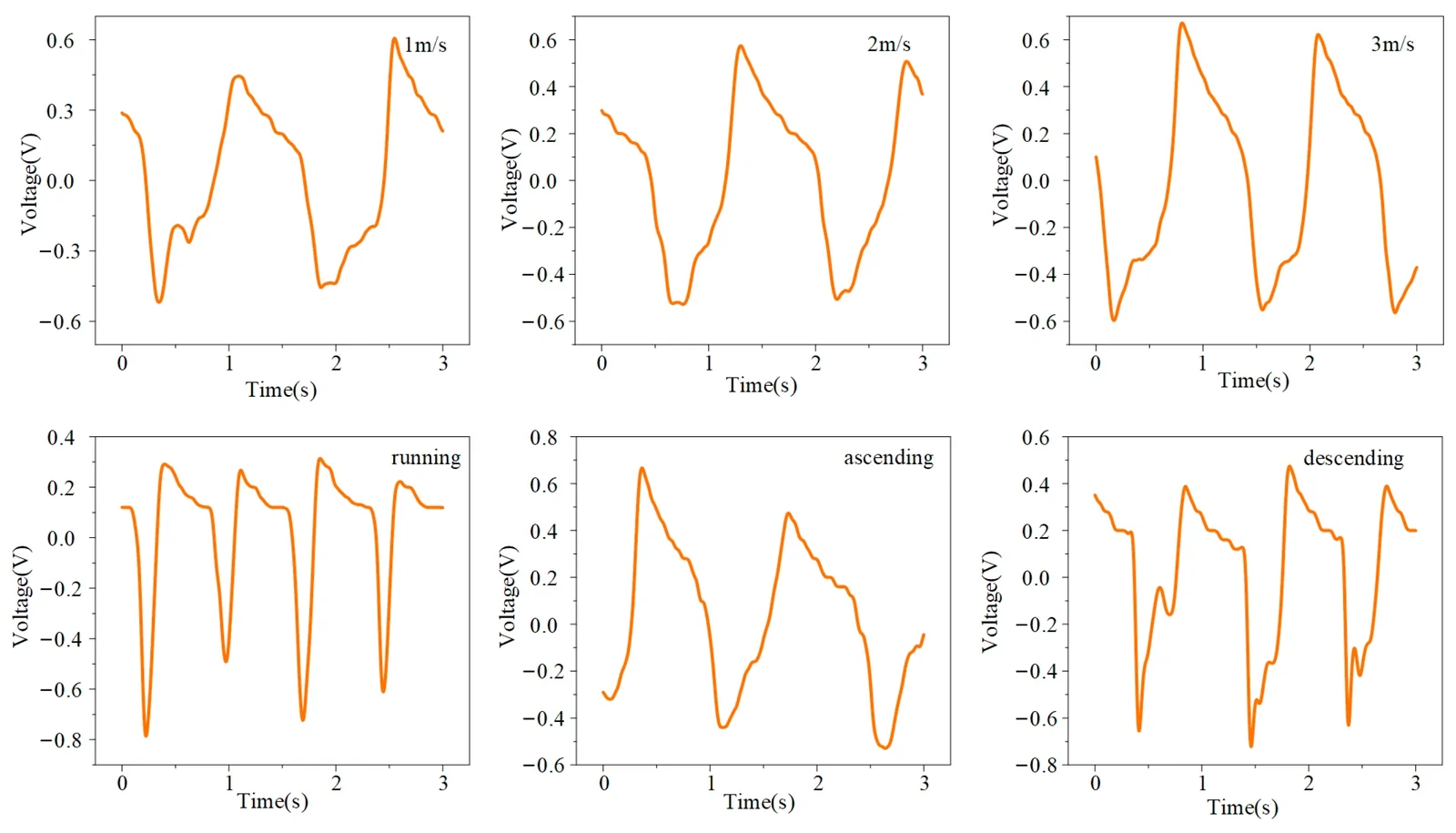
Representative piezoelectric voltage responses under six locomotion modes: walking at 1 m/s, walking at 2 m/s, walking at 3 m/s, running, ascending, and descending.

Figure 7 and Figure 8 presents representative voltage signals for walking at 1, 2, and 3 m/s, running, ascending, and descending. Each plantar loading event generated a transient voltage pulse followed by an oscillatory response.

The waveform characteristics varied with locomotion mode. As the walking speed increased, the interval between successive voltage responses decreased, while the signal amplitude and oscillation intensity generally increased. Running produced denser voltage responses because of its higher step frequency and stronger plantar impact.

Ascending and descending also exhibited different temporal and amplitude characteristics. However, their local waveform patterns showed greater similarity than those of level walking and running. Overall, the results indicate that the generated voltage contains motion-related information in addition to electrical energy. Under a running speed of 5 m/s and an external load resistance of 60 Ω, the experimentally measured maximum output power of the electromagnetic generator reached 1.053 W, providing quantitative evidence of the energy-harvesting capability of the proposed plantar device.

### 3.2. Model Training Performance

**Figure 9:**
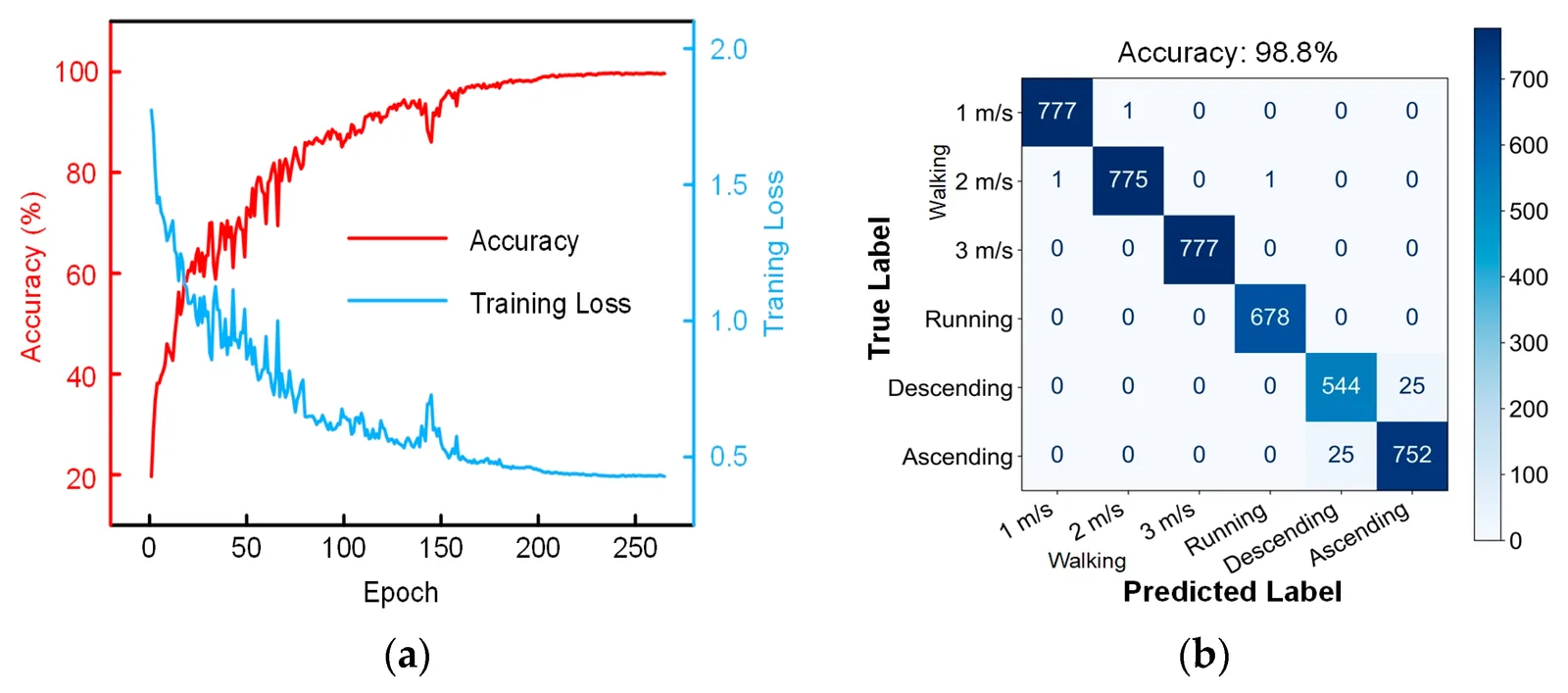
Training and classification performance of the CNN–LSTM model: (**a**) training accuracy and loss curves; (**b**) confusion matrix for the six locomotion modes.

The training performance of the CNN–LSTM model is presented in Figure 9a. The classification accuracy increased rapidly during the early training stage, while the loss decreased correspondingly. Both curves gradually stabilized as training proceeded, indicating effective convergence of the model.

The CNN extracted local waveform characteristics, while the LSTM captured the temporal dependencies of the voltage sequences. The stable convergence demonstrates that the generated voltage signals contain learnable features for locomotion recognition.

### 3.3. Locomotion Recognition Results

The confusion matrix of the six-class locomotion-recognition task is shown in Figure 9b. The CNN–LSTM model achieved an overall recognition accuracy of 98.8% for the six locomotion modes.

Walking at 1, 2, and 3 m/s was recognized with accuracies of approximately 99.87%, 99.74%, and 100%, respectively. All running samples were correctly classified. These results demonstrate that changes in walking speed and gait frequency can be distinguished from the generated voltage signals.

Most classification errors occurred between ascending and descending. Their recognition accuracies were approximately 96.78% and 95.61%, respectively, with 25 samples from each class misclassified as the other. This confusion was mainly attributed to the similar vertical loading and foot–stair contact characteristics of the two activities.

Overall, 4303 of 4356 test samples were correctly classified. To further examine subject-independent performance, LOSO cross-validation was conducted. The recognition accuracies for the three held-out participants were 98.2%, 98.4%, and 97.5%, respectively, corresponding to an average accuracy of 98.0 ± 0.39%. These results provide preliminary evidence that the model captures locomotion-related waveform characteristics rather than solely participant-specific patterns. Nevertheless, the small cohort size limits broader population-level generalization. The results demonstrate that the generated voltage can simultaneously serve as an electrical output and a sensing signal, enabling energy harvesting and locomotion recognition using the same plantar device.

To assess whether the CNN–LSTM architecture provides an appropriate balance between recognition performance and computational complexity, additional benchmark experiments were conducted using MLP, CNN, LSTM, and GRU models under the same data-partitioning protocol. Accuracy, F1-score, parameter count, and inference time were used as evaluation metrics. The comparative results are summarized in Table 1.

## 4. Discussion

The experimental results demonstrate that the proposed plantar device can integrate biomechanical energy harvesting and locomotion recognition within a single mechanical–electrical platform. The wedge–lever mechanism converts vertical plantar displacement into rotational motion, while the multistage gear train increases the generator speed. This transmission process enables low-frequency foot loading to produce measurable voltage responses during different locomotion activities.

The generated voltage is not only an electrical output but also a motion-dependent sensing signal. Changes in walking speed, impact intensity, contact duration, and gait frequency alter the amplitude and temporal distribution of the voltage waveform. Consequently, the voltage responses generated during walking, running, ascending, and descending contain distinguishable characteristics. This dual use of the generator signal avoids the need for an additional pressure sensor or inertial measurement unit and reduces the number of sensing components required in the wearable system.

The CNN–LSTM model achieved an overall recognition accuracy of 98.8%, confirming that the voltage signals contain sufficient information for locomotion classification. The high recognition accuracy for level walking and running indicates that the model can effectively distinguish changes in gait frequency and loading intensity. In comparison, most errors occurred between ascending and descending because these two activities involve similar vertical loading and foot–stair contact processes. Their differences are mainly reflected in more subtle temporal and waveform characteristics, making them more difficult to separate.

The proposed approach provides a compact route toward the integration of energy harvesting and self-sensing in smart footwear. Unlike conventional wearable monitoring systems that rely on separately powered sensors, the present device obtains both electrical energy and locomotion information from the same plantar excitation. This configuration may reduce system complexity and provide useful motion information for activity monitoring, rehabilitation assessment, and human–machine interaction.

Several limitations should nevertheless be considered. First, the current experiments involved only three healthy participants under controlled laboratory conditions, and the generalization capability of the recognition model should be further evaluated using a larger and more diverse population. Second, the present prototype requires further optimization in terms of thickness, mass, structural durability, and wearing comfort. Third, the present study focused on demonstrating the integrated energy-harvesting and self-sensing capability. Although a maximum output power of 1.053 W was experimentally measured under a 60 Ω load at a running speed of 5 m/s, synchronized force–displacement measurements were not performed. Therefore, harvested energy per gait cycle and the overall mechanical-to-electrical conversion efficiency were not quantitatively evaluated. Future work will address these metrics through load optimization and comprehensive energy-conversion tests. At the present stage, the recognition model is operated offline, and the system should therefore be regarded as an integrated harvesting–sensing prototype rather than a fully energy-autonomous wearable platform.

## 5. Conclusions

This study proposed a gear-driven plantar energy harvester with integrated self-sensing capability. The device converts low-frequency vertical plantar loading into high-speed generator rotation through a wedge–lever mechanism, a one-way bearing, and a multistage gear train with a total speed-amplification ratio of 12. The generated voltage can be used both as an electrical output for energy harvesting and as a sensing signal for locomotion recognition.

Experiments were conducted under six locomotion modes: walking at 1, 2, and 3 m/s; running; ascending; and descending. The voltage signals exhibited mode-dependent differences in amplitude, temporal distribution, and oscillatory characteristics. Based on these signals, the CNN–LSTM model achieved an overall recognition accuracy of 98.8%, with most classification errors occurring between ascending and descending.

The results demonstrate the feasibility of integrating biomechanical energy harvesting and locomotion recognition within a single plantar device. This approach reduces the dependence on additional sensing components and provides a potential solution for integrated energy-harvesting and self-sensing wearable systems, activity monitoring, and human–machine interaction.

## Figures and Tables

**Table 1:** Benchmark comparison of different locomotion-recognition models.

Model	Accuracy	F1-Score	Parameters (M)	Inferences (ms)
MLP	0.615	0.611	2.16	0.140
CNN	0.951	0.938	0.04	0.200
LSTM	0.698	0.698	0.20	6.93
GRU	0.940	0.911	0.15	2.22
CNN-LSTM	0.978	0.967	0.26	6.94

## Data Availability

The data presented in this study are available on request from the corresponding author.

## References

[1] Tang W., Sun Q., Wang Z.L. (2023). Self-Powered Sensing in Wearable Electronics—A Paradigm Shift Technology. Chem. Rev..

[2] Yao Z., Guan Z., Zhang S., Zeng J., Zhang X., Wu Z., Hu Z. (2025). Towards Energy-Autonomous Wearables: Multimodal Harvesting Technologies and Sustainable Applications. Innov. Mater..

[3] Sharghi H., Bilgen O. (2022). Energy Harvesting from Human Walking Motion Using Pendulum-Based Electromagnetic Generators. J. Sound Vib..

[4] Liu M., Qian F., Mi J., Zuo L. (2022). Biomechanical Energy Harvesting for Wearable and Mobile Devices: State-of-the-Art and Future Directions. Appl. Energy.

[5] Li X., Zeng X., Li J., Li B., Chen Y., Zhang X. (2024). Biomechanical Energy Harvesting Technologies for Wearable Electronics: Theories and Devices. Friction.

[6] Laganà F. (2026). Design and Simulation-Based Validation of an Embedded Acquisition Architecture for In Situ PCB Integrity Monitoring in Biomedical Devices. Electronics.

[7] Zheng Q., Dai X., Wu Y., Liang Q., Wu Y., Yang J., Dong B., Gao G., Qin Q., Huang L. (2023). Self-powered High-resolution Smart Insole System for Plantar Pressure Mapping. BMEMat.

[8] Xu J., Li H., Yin Y., Li X., Cao J., Feng H., Bao W., Tan H., Xiao F., Zhu G. (2022). High Sensitivity and Broad Linearity Range Pressure Sensor Based on Hierarchical In-Situ Filling Porous Structure. npj Flex. Electron..

[9] Parashar P., Sharma M.K., Nahak B.K., Khan A., Hsu W.-Z., Tseng Y.-H., Chowdhury J.R., Huang Y.-H., Liao J.-C., Kao F.-C. (2025). Machine Learning-Driven Gait-Assisted Self-Powered Wearable Sensing: A Triboelectric Nanogenerator-Based Advanced Healthcare Monitoring. J. Mater. Chem. A.

[10] Li X., Liu Y., Ding Y., Zhang M., Lin Z., Hao Y., Li Y., Chang J. (2024). Capacitive Pressure Sensor Combining Dual Dielectric Layers with Integrated Composite Electrode for Wearable Healthcare Monitoring. ACS Appl. Mater. Interfaces.

[11] Zhou X., Parida K., Chen J., Xiong J., Zhou Z., Jiang F., Xin Y., Magdassi S., Lee P.S. (2023). 3D Printed Auxetic Structure-Assisted Piezoelectric Energy Harvesting and Sensing. Adv. Energy Mater..

[12] Ligęza P. (2026). Emergency Power Sources Operating Based on Energy Harvesting Processes for Application in Crisis Situations. Energies.

[13] Xiang K., Liu M., Chen J., Bao Y., Wang Z., Xiao K., Teng C., Ushakov N., Kumar S., Li X. (2024). AI-Assisted Insole Sensing System for Multifunctional Plantar-Healthcare Applications. ACS Appl. Mater. Interfaces.

[14] Santos V.M., Gomes B.B., Neto M.A., Rodrigues P.F., Amaro A.M. (2025). A Systematic Review on Smart Insole Prototypes: Development and Optimization Pathways. Actuators.

[15] Cudejko T., Button K., Al-Amri M. (2023). Wireless Pressure Insoles for Measuring Ground Reaction Forces and Trajectories of the Centre of Pressure during Functional Activities. Sci. Rep..

[16] D’Arco L., Wang H., Zheng H. (2025). Integration of Smart Insoles for Gait Assessment in Exoskeleton Assisted Rehabilitation. Sci. Rep..

[17] Sun M., Cui S., Wang Z., Luo H., Yang H., Ouyang X., Xu K. (2024). A Laser-Engraved Wearable Gait Recognition Sensor System for Exoskeleton Robots. Microsyst. Nanoeng..

[18] Lee J., Lee J., Lee Y.J., Kim H., Kwon Y., Huang Y., Kuczajda M., Soltis I., Yeo W.-H. (2025). Flexible Smart Insole and Plantar Pressure Monitoring Using Screen-Printed Nanomaterials and Piezoresistive Sensors. ACS Appl. Mater. Interfaces.

[19] Lugoda P., Hayes S.C., Hughes-Riley T., Turner A., Martins M.V., Cook A., Raval K., Oliveira C., Breedon P., Dias T. (2022). Classifying Gait Alterations Using an Instrumented Smart Sock and Deep Learning. IEEE Sens. J..

[20] Tian Y., Zhang L., Zhang C., Bao B., Li Q., Wang L., Song Z., Li D. (2024). Deep-learning Enabled Smart Insole System Aiming for Multifunctional Foot-healthcare Applications. Exploration.

[21] Wang Q., Guan H., Wang C., Lei P., Sheng H., Bi H., Hu J., Guo C., Mao Y., Yuan J. (2025). A Wireless, Self-Powered Smart Insole for Gait Monitoring and Recognition via Nonlinear Synergistic Pressure Sensing. Sci. Adv..

[22] Fastier-Wooller J.W., Lyons N., Vu T.-H., Pizzolato C., Rybachuk M., Itoh T., Dao D.V., Maharaj J., Dau V.T. (2024). Flexible Iron-On Sensor Embedded in Smart Sock for Gait Event Detection. ACS Appl. Mater. Interfaces.

[23] Zhang Y., Wang E., Ouyang H., Li Z. (2025). Self-Powered Wearable Sensing Devices for Digital Health. Mater. Horiz..

[24] Sun R., Wu P., Li P., Jiang J., Shou M., Chen Q., Yang P., Wang F., Liao C. (2025). Bio-Inspired Triboelectric Nanogenerator as a Self-Powered Gait Recognition Sensor for Legged Robots. Sci. China Mater..

[25] Zhang S., Li Y., Zhang S., Shahabi F., Xia S., Deng Y., Alshurafa N. (2022). Deep Learning in Human Activity Recognition with Wearable Sensors: A Review on Advances. Sensors.

[26] Khatun A., Yousuf M.A., Ahmed S., Uddin Z., Alyami S.A., Al-Ashhab S., Akhdar H.F., Khan A., Azad A., Moni M.A. (2022). Deep CNN-LSTM With Self-Attention Model for Human Activity Recognition Using Wearable Sensor. IEEE J. Transl. Eng. Health Med..

[27] Yuan H., Chan S., Creagh A.P., Tong C., Acquah A., Clifton D.A., Doherty A. (2024). Self-Supervised Learning for Human Activity Recognition Using 700,000 Person-Days of Wearable Data. npj Digit. Med..

[28] Punnarao M., Yoon H.-J. (2026). Flexible Triboelectric Mechanical Energy Harvesters for Wearable and Self-Powered Sensing Applications: A Review. Sensors.

